# Self-Assembly of Symmetric Copolymers in Slits with Inert and Attractive Walls

**DOI:** 10.3390/polym15224458

**Published:** 2023-11-18

**Authors:** Tomáš Blovský, Karel Šindelka, Zuzana Limpouchová, Karel Procházka

**Affiliations:** 1 The Department of Physical and Macromolecular Chemistry, Faculty of Science, Charles University in Prague, Hlavova 2030, 128 40 Prague, Czech Republic; blovskyt@natur.cuni.cz; 2 Department of Molecular and Mesoscopic Modelling, Institute of Chemical Process Fundamentals, Czech Academy of Sciences, Rozvojová 135, 165 02 Prague, Czech Republic; sindelka@icpf.cas.cz

**Keywords:** confined copolymer, self-assembly, selective solvent, slit, polymer–wall interaction, dissipative particle dynamics

## Abstract

Although the behavior of the confined semi-dilute solutions of self-assembling copolymers represents an important topic of basic and applied research, it has eluded the interest of scientists. Extensive series of dissipative particle dynamics simulations have been performed on semi-dilute solutions of A_5_B_5_ chains in a selective solvent for A in slits using a DL-MESO simulation package. Simulations of corresponding bulk systems were performed for comparison. This study shows that the associates in the semi-dilute bulk solutions are partly structurally organized. Mild steric constraints in slits with non-attractive walls hardly affect the size of the associates, but they promote their structural arrangement in layers parallel to the slit walls. Attractive walls noticeably affect the association process. In slits with mildly attractive walls, the adsorption competes with the association process. At elevated concentrations, the associates start to form in wide slits when the walls are sparsely covered by separated associates, and the association process prevents the full coverage of the surface. In slits with strongly attractive walls, adsorption is the dominant behavior. The associates form in wide slits at elevated concentrations only after the walls are completely and continuously covered by the adsorbed chains.

## 1. Introduction

The diverse behaviors of block copolymers have been attracting the attention of polymer chemists and physicists for many years. In selective solvents (a good solvent for some blocks and poor for the other blocks), block copolymers self-assemble and form various nanoparticles (core–shell micelles, multicompartment micelles, vesicles, etc.) depending on the solvent’s selectivity, chain architecture (number and relative lengths of individual blocks), stiffness of the blocks, and other factors. Self-assembly is a result of the interplay between the interaction enthalpy of the components (block-building units and solvent) and the entropy of frustrated incompatible blocks, which cannot macroscopically separate due to their covalent connection. In the overwhelming majority of cases, the process is enthalpy-driven and entropy-controlled [1,2]. In aqueous systems, the hydrophobic effect and changes in water structure play important roles, and the assembly can also be slightly favorized by the entropy [3]. Thanks to promising applications of self-assembled nanoparticles in various biomedical and technological fields (nanocontainers, drug delivery systems, biomarkers and biosensors, bioactive agents, pollutant removals, photoelectric materials, catalysts, etc.) [4,5,6,7,8], high numbers of self-assemblies have been prepared and studied, and the association process and the behavior of nanoparticles in bulk solvents are fairly well understood [9,10,11,12,13,14,15,16,17,18,19,20,21,22].

In recent years, the interest of scientists and engineers has shifted towards self-assembly in confined volumes [23,24,25,26,27] because this topic is important for separation and chromatographic studies of polymers, the development of novel tunable structures (unachievable in bulk), etc. Concerning the latter topic, self-assembly in emulsion droplets, also called evaporation-induced confinement assembly (EICA), has proven to be a very versatile and promising method for the fabrication of targeted nanostructures [28,29,30,31]. As already mentioned, the area of constrained polymer self-assembly is broad and also comprises the separations and chromatographic studies of associating polymer systems [32] and the behavior of various 2D and 1D systems, including the practically significant topics of structured thin films [33,34,35,36] and tethered chains [37,38,39,40].

A literature search revealed that researchers have been studying either strongly geometrically restricted polymer and copolymer dilute solutions [32,41,42,43,44,45,46] or concentrated systems submitted to strong 3D confinements [28,47,48,49], and mildly confined and medium concentrated systems have eluded the interest of both experimentalists and theoreticians. Thus, in this paper, we investigate the behavior of weak to medium-strong confined semi-dilute self-assembling copolymer systems and study the onset of constraint-induced and adsorption-induced structural changes.

## 2. Methodology

### 2.1. Simulation Method

All simulations were performed using dissipative particle dynamics, DPD, which is a coarse-grained variant of the molecular dynamics method developed by Hoogerbrugge and Koelman for studying large systems [50] and has been further modified by Groot, Warren, Español, and others [51,52,53,54,55,56]. The principles and methodology of DPD have been recently described in excellent review articles by Santo and Neimark [51] and Zhao [57], and the perspectives of the coarse-grained approaches towards colloid and polymer self-assembly were outlined in a review article by Lombardo et al. [58]. In DPD, the studied species are represented by soft DPD beads. The beads of low-molar-mass compounds (e.g., solvents and monomers) actually comprise several molecules. The polymers and oligomers are modeled as a string of several interconnected beads. The force acting between beads is expressed as a sum of three contributions: (i) a soft conservative force, $F^{C}=-\nabla_{r}u^{\mathrm{CG}}$, derived from the coarse-grained inter-bead potential, $u^{\mathrm{CG}}$, describing the interaction between the coarse-grained beads; (ii) a dissipative force, ***F*^D^**, reflecting the friction that slows down the average motion of beads; and (iii) a random force, ***F***^R^, emulating thermal agitation and intermolecular collisions, which accelerate the particles. The forces ***F*^D^** and ***F***^R^ substitute high numbers of degrees of freedom neglected by the coarse-graining approach in favor of fast simulations. All forces are spatially limited, and their cutoff distance defines the size of the DPD beads and serves as the length unit. To secure a constant total energy during the simulation run, ***F*^D^** and ***F***^R^ have to be well balanced. The dissipative fluctuation theorem applied in DPD by Español and Warren [56] secures the appropriate balance and serves as a sort of thermostat.

The effective pair interactions acting between beads *i* and *j* are “soft” and do not diverge at short distances *r_ij_*, which means that the DPD beads also behave as “soft” objects and can interpenetrate each other. The conservative force includes the (i) soft and spatially limited non-electrostatic repulsion, (ii) electrostatic interaction between charged particles, if the system contains the electrically charged particles, and (iii) bonding force, if the polymers, surfactants, or larger molecules (e.g., porphyrins) are present [59,60,61,62,63]. Flexible polymer chains are modeled as strings of interacting beads connected by elastic springs (usually described by the harmonic spring potential with the spring constant *K* and the equilibrium distance *r*_0_ between two neighboring beads), emulating the covalent bonds.

In DPD, all non-electric forces are repulsive, and their parameters are calibrated on the basis of the compressibility vs. density relationship (equation of state). This means that a weaker repulsion between some components than those acting between other components represents relative attraction. This treatment is analogical to the Flory–Huggins theory (FH) of concentrated polymer solutions, which also employs the relative comparison of the strength of interactions. In the FH theory, various thermodynamic characteristics are expressed as functions of the interaction parameter, *χ_ij_*, which is based on the difference between the cross-interaction and the average of the homo-interactions of the components [64]. In fact, the DPD parameters of soft repulsive forces, *a_ij_*, can be readily recalculated in the Flory–Huggins interaction parameters, *χ_ij_*. Groot and Warren [55] mapped DPD results onto FH systems and established a reasonable link between the above parameters. Using the appropriate equation of state for the soft repulsive DPD fluid together with the value of compressibility for ambient water, and assuming *a_ii_* = *a_jj_* they derived for polymer systems $\rho r_{c}^{3}=3$ the following simple relation:

(1) $$\frac{a_{ij\;}r_{c}}{kT}=\frac{a_{ii\;}r_{c}}{kT}+3.27\;\chi_{ij}\;$$

It is worth mentioning that the interactions between molecules of the same compound or between compatible components, e.g., between DPD beads of a well-soluble polymer and solvent, described by *χ* = 0, translate into *a_ij_* = 25. The value *a_ij_* = 26.64 corresponds to the *θ*-state (*χ* = ½), and *a_ij_* = 40 describes strongly unfavorable interactions corresponding to *χ* = 4.59. Values lower than *a_ij_* = 25 describe a significant attraction. In this study, we studied electrically neutral (non-charged) systems, i.e., electrostatic interactions were not taken in account.

In the simulations, the Newtonian equations of motions for a system of many DPD beads subjected to inter-particle forces are numerically solved, and the structural and thermodynamic characteristics of the system are evaluated and averaged during the simulation runs. The pair-wise nature of dissipative and random forces ensures the correct hydrodynamic behavior because the momentum is locally conserved.

### 2.2. Simulation Details

We studied the behavior of a block copolymer in a solvent, S, which is good for A beads and poor for B beads. The copolymer chains were modeled as flexible strings of 5 well-soluble beads, A, and 5 insoluble beads, B, A_5_B_5_, connected by harmonic strings with *K* = 4 and *r*_0_ = 0. The interaction parameters were set based on our earlier DPD studies of copolymer self-assembly and are summarized in Table 1 [56,60,65,66,67]. The coefficient of the stochastic force was set to *σ_ij_* = 3, and the friction coefficients were *γ_ij_* = 4.5. The numbers of A_5_B_5_ chains in bulk ranged from 120 to 1500 to match the required concentration. The numbers of chains in slits of a given size were adjusted according to the actual volumes to secure the required concentration.

The simulations of bulk copolymer solutions were performed in a cubic 25^3^ simulation box with the periodic boundary conditions applied in all 3 dimensions. The simulations in slits were performed in rectangular *D* × 25^2^ simulation boxes, with *D* ranging from 10 to 25, and with boundary conditions applied in the directions of the *y-* and *z*-axes. The concentration is expressed as the effective volume fraction of polymer DPD beads (in reduced units—see below) to the volume of the simulation box, *V* (e.g., *V* = 25^3^, for bulk). However, as all DPD beads (polymer beads, as well as those of the solvent) are soft and can mutually interpenetrate, and the DPD simulations are commonly performed at densities of *ρ >* 1, the formula for the volume concentration (in vol. %) reflects the softness of the particles and the applied density and reads: *c =* 100(*Nn/ρV*), where *N* is the number of copolymer chains in the simulation box and *n* = 10 is the number of DPD beads in one chain. For simplicity, in the next sections, we use the shortened symbol %.

The slit walls are perpendicular to the *x*-axes and are modeled as layers of frozen beads that do not change their positions during the simulation. The wall beads interact with each other and with all other particles in the system by soft repulsion with the parameters summarized in Table 1. We used hard reflecting boundaries (the velocity component normal to the wall of any particle leaving the system at a particular boundary is inverted, and the tangential velocity components remain the same) in combination with short-range soft repulsive potential in the form

(2) $$\begin{array}{lll} U_{k,wall}^{W} & =\frac{a_{i,wall}}{2}\;x_{c}\;\left(1-\frac{x_{k,wall}}{x_{c}}\;\right)\; & \left(x_{k,wall}<x_{c}\right)\\ & =0 & \left(x_{k,wall}>x_{c}\right) \end{array}$$

where *a_k_*_,wall_ is the maximum repulsion between *k*-particles and the wall, and *x*_c_ defines the surface repulsion range [68]. According to reference [69], we set the parameter values *a_k_*_,wall_ = 25 and *x*_c_ = 1.2 to suppress the density oscillations in the vicinity of the wall.

**Scheme 1:**
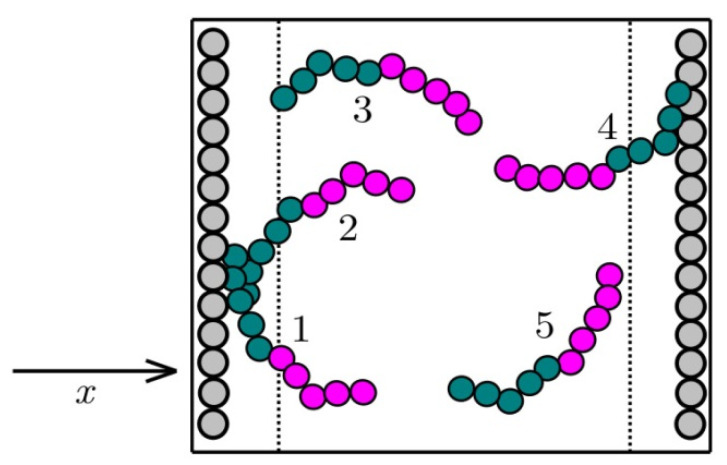
Polymer 1 has three contacts of B blocks (cyan) with the wall (gray), and therefore is classified as polymer adsorbed on the wall. Polymer 2 is in contact with polymer 1, and thus is also classified as adsorbed polymer. Polymer 4 is adsorbed on the wall. Polymers 3 and 5 are in the slab. The soluble blocks A are marked by purple color.

For the aggregate definition, we used the most commonly used criterion based on the contacts between the core-forming beads from different polymers. Two beads from different chains form a contact pair if their distance is lower than the interaction cutoff. Two polymers belong to the same aggregate if they have at least three contact pairs of solvophobic B beads between two different polymer chains. We tested the criteria based on 1–4 numbers of contact pairs, and the three-contact criterion was chosen on the basis of a visual inspection of a large collection of snapshots. The three-contact criterion is also used for the definition of adsorbed polymers: the aggregate or polymer is adsorbed on the wall if it contains at least one chain with three B beads in contact with the wall; see Scheme 1. The aggregates or polymers not adsorbed on the wall (i.e., polymers that are not in contact with the wall or in contact with other adsorbed polymers) are classified as non-adsorbed polymers inside the box (free chains in the slab).

We used reduced units: the unit of length equals the interaction cutoff, *r*_c_, and the unit of energy equals the product of the Boltzmann constant and the thermodynamic temperature, k*T*. Further for simplicity, we assumed that all beads had the same mass, and this mass served as the unit of mass, *m*_0_. The time step was 0.05 reduced units, *τ* = (*mr*_c_^2^/k*T*)^1/2^. The individual simulation runs for the bulk systems consisted of a 10^7^-simulation-steps-long pre-equilibration period and a 7 × 10^7^-simulation-steps-long simulation run. For the slit simulations, the pre-equilibration part consisted of 10^7^ simulation steps, and the simulation run consisted of approximately 5 × 10^7^–7 × 10^7^ simulation steps. The period of 10^4^ simulation steps was set to generate independent configurations for averaging the simulation data, and 5 × 10^3^ to 10 × 10^3^ configurations were averaged in general.

## 3. Results and Discussion

### 3.1. Unconfined Semi-Dilute Solutions

In order to evaluate the impact of steric constraints and correctly estimate the onset of the constraint-induced changes in slits of different widths at different copolymer concentrations, the behavior of the corresponding unconstrained system (described by an identical set of parameters) has to be known in detail. Therefore, we first performed simulations of the self-assembly in bulk solutions in a relatively wide range of concentrations from ca. 6 to 30%. These simulations revealed general trends of the behavior and simultaneously provided proof that the self-assembling copolymer systems in the selective solvent used (which was fairly poor for block B) did not freeze and that the associates coexisted in a mobile equilibrium with single chains (unimers). Note that we address relatively concentrated systems.

**Figure 1:**
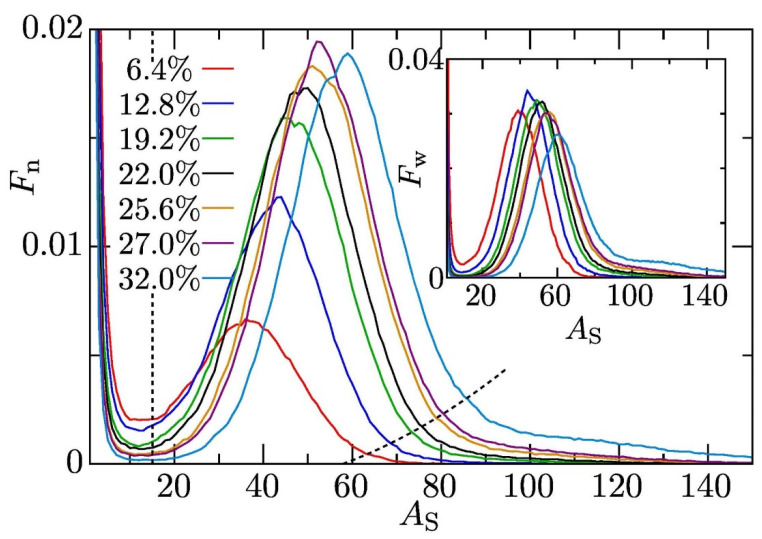
Number distribution functions, *F*_n_(*A*_S_), of associates formed at different concentrations (including unimers). Insert: the corresponding weight distribution functions *F*_w_(*A*_S_). The dashed curves indicate the lower and upper limits of associates included in the main peak at given concentrations (see the explanatory text in the preceding paragraph).

In Figure 1, we present the number and weight distribution functions, *F*_n_(*A*_S_) and *F*_w_(*A*_S_), of the association numbers of associates formed in bulk at different concentrations. The simulation data clearly show that the used set of interaction parameters models the behavior of A_5_B_5_ copolymers in a medium-strong selective solvent for block A. The multichain associates formed in the whole range of the studied concentrations and coexisted in equilibrium with unimers. The number fractions of unimers were significant (particularly in relatively dilute systems), but the fractions of low associates dropped fast with the increasing association number *A*_S_. The curves pass the minima at *A*_S_ ca. 10–15, and the well-pronounced peaks of associates are well separated from the peak of the unimers (and low associates) in all systems. With an increasing copolymer concentration, the fraction of associates grew, and the peak of associates shifted to a higher *A*_S_, i.e., the average association number increased. To simplify the description and discussion of the simulation results, we tentatively divided the distributions of the association numbers into the main peak and the tail. The division (marked by a smooth dotted line in Figure 1) is based on the maxima of second derivatives of the individual distribution functions.

**Figure 2:**
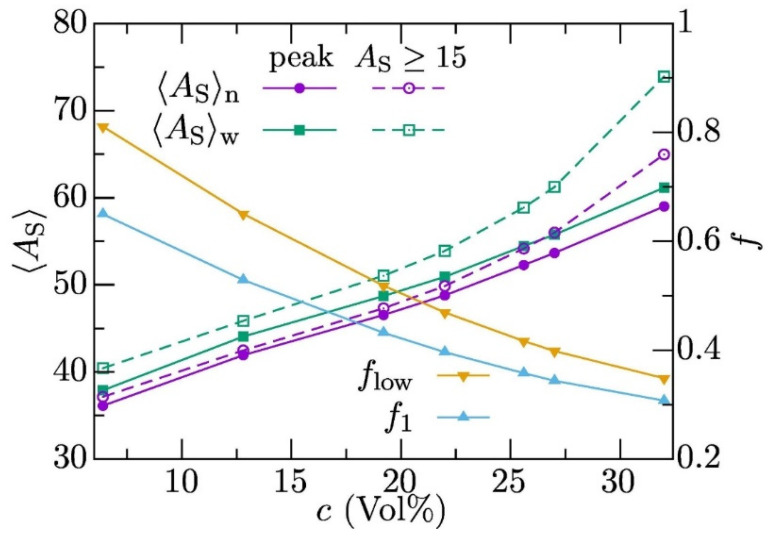
Number-average and weight-average association numbers of associates (left scale), and the number fraction of unimers, *f*_1_, and unimers together with low associates, *f*_low_ (up to the minima at the distribution functions, right scale), as a function of the concentration, *c*. Solid lines correspond to the associates from the main peak of the distribution and the dashed lines to the associates with *A*_s_ ≥ 15.

Figure 2 shows the dependences of the number-average and weight-average association numbers of associates and the number fraction of either neat unimers, *f*_1_, or unimers together with low associates (up to the minima at the distribution functions), *f*_low_, on the concentration of copolymer chains in the system. As already mentioned, the number fractions of unimers and low associates were fairly high in the dilute systems and decreased with increasing concentration, but they were still significant at the highest concentration studied. The weight fractions of unimers were low in all cases (invisible at the scale used) and vanished at high concentrations.

According to the closed association scheme, which represents the generally recognized model of the self-assembly of well-defined high-molar-mass diblock copolymers, the concentration of unimers should remain constant above a critical micelle concentration, the distribution of the *A*_S_ of associates should be fairly narrow, and the average association number should not depend on the copolymer concentration. However, it is a generally accepted fact that, in contrast to the behavior of macroscopic solutions of high-molar-mass copolymers studied experimentally, computer simulations yield broader concentration-dependent *A*_S_ distributions due, in part, to relatively short lengths of simulated chains and to the significantly restricted size of studied systems [62,70]. Moreover, the closed association scheme was proposed and tested for very dilute solutions of long chains only.

Inspection of the distributions of the association numbers in Figure 1 reveals pronounced tails towards a high *A*_S_, particularly in solutions with concentrations approaching and exceeding 20%. We assumed that the main peak corresponds to roughly spherical core–shell micelles and the tail to non-spherical associates. This working hypothesis is based on the following arguments. The radius of spherical cores is controlled by the length of the insoluble block and is restricted because the formation of spherical cores exceeding the optimum size would require enthalpically unfavorable intermixing of both blocks. Hence, the large associates are not spherical core–shell micelles but rod-like objects, the diameter of which is controlled by the length of the insoluble block, and their length is not restricted. To prove this hypothesis, we analyzed the shapes of associates differing in *A*_S_ and present typical snapshots of the simulation box.

**Figure 3:**
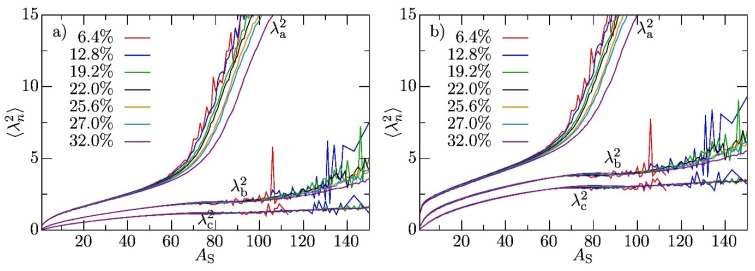
Diagonal components of the gyration tensor of (**a**) insoluble cores of associates and (**b**) whole associates as functions of *A*_S_.

**Figure 4:**
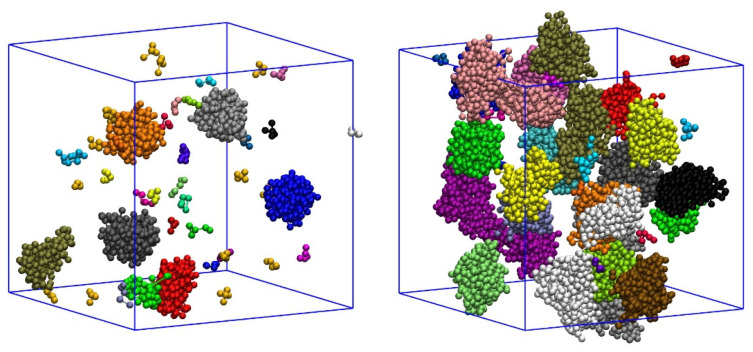
Randomly chosen snapshots at concentrations of 6.4% and 32%. For clarity, only the aggregate cores (B beads) are shown, and individual cores are shown in different colors. The same type of color distinction of the different associates and their cores is used in all snapshots in the rest of the paper.

Figure 3a shows the diagonal components of the gyration tensor of the insoluble cores of associates for systems differing in copolymer concentration as functions of *A*_S_, and Figure 3b shows the corresponding data for whole associates. The data show that the associates with *A*_S_ in the region of the main peak adopted slightly prolate ellipsoidal shapes but did not appreciably differ from spheres (all three components are comparable); see also the corresponding snapshots in Figure 4. It is worth mentioning that the sizes of associates with the given *A*_S_ are concentration-independent and that the slowly growing convex curves of squares of size parameters *λ_i_*^2^ vs. *A*_S_ in the region of the main peak, i.e., *λ_i_* ∝ (*A*_S_)*^a^*, with 0 < *a* < 1, indicate the formation of relatively compact objects. Note that the effective radius of a homogeneous spherical particle is proportional to the third root of its mass (in our case, the association number), i.e., *a* = 1/3.

In the region of high *A*_S_, the concentration-independent short and medium components of the gyration tensor continued in the slow growth with *A*_S_, but the longest concentration-dependent components (both of the cores and associates) increased fast. This indicates that the species in the tails of *F*_w_(*A*_S_) differed significantly in shape from the associates in the main peak region. This, together with a vast collection of snapshots (two of them shown in Figure 4), confirms that a fraction of the prolate core–shell particles of almost constant diameter and significantly varying length formed at elevated concentrations.

The reason a fraction of large rod-like associates forms and coexists with regular spherical micelles in concentrated systems can be rationalized as follows: Micelles are fairly crowded at elevated concentrations, and the repulsion between their shells plays an important role [71]. A repulsive interaction occurs when the shells of aggregates approach each other and the parts of their uppermost surfaces interact. Because the surface-to-volume ratio generally decreases with the size of aggregates, the repulsion at a given concentration of the aggregating copolymer chains is less important in systems with a lower number of larger associates, which, as explained above, have to adopt a prolonged shape. Moreover, the cylindric associate with diameter *D* and length *ND* can accommodate 1.5 times more polymer chains than *N* spheres of diameter *D*, which significantly contributes to the dilution of aggregates.

Further, we investigated how the elevated concentration affects the organization of micelles in bulk solutions. It is a well-recognized fact that a lamellar structure is the typical organization of the melt of symmetric high-molar-mass diblock copolymers. The associates in the studied solutions were crowded, but their concentrations were far from that in the melt. Therefore, it is of interest to study the onset and gradual development of the concentration-dependent organization of associates in space. As the formation of organized structures (lamellas, hexagonal phase, or other structural motifs, both perfect and imperfect) generates oscillations in the local density of polymer blocks, the scans of the average density of individual blocks in properly selected directions should reveal the spatial orientation. The results of extensive auxiliary studies are shown and discussed in the Supplementary Materials (SMs). The data in Figures S1 and S2 show the imperfect (partial) organization of associates in semi-dilute bulk solutions and simultaneously provide unambiguous proof that the studied systems are not kinetically frozen.

### 3.2. Sterically Confined Self-Assembling Systems in Slits

The behavior of self-assembling block copolymers in slits is complex, and its unambiguous and simultaneously straightforward description revealing and clearly depicting the most important trends of the behavior is complicated. In slits with attractive walls, the structures adsorbed on walls can differ from the associates formed in the central part of the slit. Depending on the slit width, the copolymer concentration, and the relative strength of competing interactions promoting either the self-assembly or the adsorption of copolymer chains on walls, the polymers can be adsorbed on the wall as (i) a fairly homogeneous or inhomogeneous layer of unimers, (ii) separated irregular islands of loosely/densely interconnected associates, or (iii) individual (separated) deformed pancake-like micelles. It is obvious that the functions used in the previous part are useful but do not provide full information about the system behavior. Therefore, the analysis of the simulation trajectories and the ensemble-averaged characteristics was supplemented by typical snapshots.

The next part is divided into two subchapters devoted to slits with (i) non-attractive walls and (ii) attractive walls. The inert (non-attracting) wall is characterized by the interaction parameters *a*_AW_ = *a*_BW_ = *a*_AA_ = *a*_BB_ = 25, i.e., the interactions of both blocks with the wall are the same as the homo-interactions and interactions of polymer beads with a good solvent. This set of parameters prevents the interaction (both attraction and repulsion) with the wall if the copolymer is dissolved in a good common solvent. However, the solvent used was not a good common solvent. It was a selective solvent for A beads, and its interaction with B beads was unfavorable. In our recent studies [32], we found that, in this case, block B preferred the interaction with the wall to that with the solvent, and it slightly adsorbed on the wall (the number of adsorbed chains was low, but the adsorbed fraction was non-negligible at low concentrations). Therefore, we supplemented our study by simulations in slits with slightly repulsive walls, for *a*_BW_ = 30, and started the next part with this slightly repulsive system.

### 3.3. Self-Assembly in Slits with Inert and Slightly Repulsive Walls

**Figure 5:**
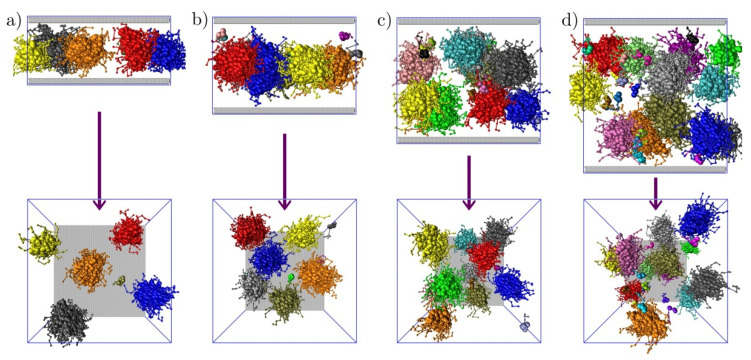
Randomly chosen snapshots of associates formed in slits with slightly repulsive walls, *a*_BW_ = 30, and widths (**a**) *D* = 10, (**b**) 15, (**c**) 20, and (**d**) 25 at a relatively low concentration of 12.8%. For clarity, the individual aggregates are labeled with different colors. The B beads are shown as balls and the A beads as thin rods. The top row provides the views inside the slits in the *z* direction (i.e., rectangular to the *xy* plane), and the bottom row contains the corresponding perspective views through the wall (along the *x*-axis, i.e., rectangular to the *yz* plane). The arrows indicate the direction of the view (through the wall) with respect to the upper panel.

To provide the reader with an idea of the behavior of studied systems in slits with slightly repulsive walls and to facilitate a subsequent discussion of the simulation results, we present typical snapshots of the simulation box first. Figure 5 illustrates the behavior of a relatively dilute system (*c* = 12.8%) in slits with increasing widths (*D* = 10, 15, 20, and 25). The top row provides the rectangular top-to-bottom views inside the slits in the *z* direction (i.e., rectangular to the *xy* plane), and the bottom row offers the corresponding perspective views through the wall (along the *x*-axis, i.e., view rectangular to the *yz* plane). The arrows indicate the direction of the view (through the wall) with respect to the upper panel.

The upper row shows that at a relatively low concentration (*c* = 12.8%), the centers of the associates are preferentially located in the middle of the narrow slits (*D* = 10 and 15), i.e., they lay in the plane (*x* = 0, *z*) parallel to the walls. The wider slits provide more space and freedom, and the centers are less regularly arranged, but the structural arrangement is still obvious. In slits with *D* = 20 and 25, we see two and three imperfect planar layers, respectively. The bottom panels show that the *y* and *z* positions of the associates in layers are fairly random.

Careful inspection of a large collection of snapshots reveals that neither the associates nor the unimers (a low fraction coexisting in equilibrium with the associates) adhered to the wall. Only in a tiny fraction of snapshots, one or two polymer beads collided with the wall as a result of thermal motion.

**Figure 6:**
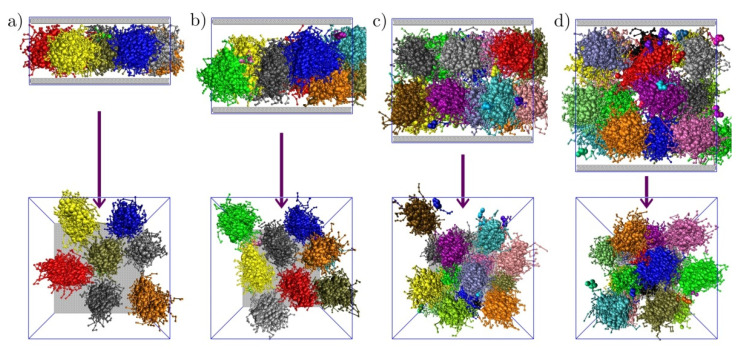
Randomly chosen snapshots of aggregates formed in slits with slightly repulsive walls, *a*_BW_ = 30, and widths (**a**) *D* = 10, (**b**) 15, (**c**) 20, and (**d**) 25 at the elevated concentration 25.6%. For clarity, the individual aggregates are labeled by different colors. The B beads are shown as balls, and the A beads as thin rods. The top row provides views inside the slits in the *z* direction (i.e., rectangular to the *xy* plane), and the bottom row contains the corresponding perspective views through the wall (along the *x*-axis, i.e., rectangular to the *yz* plane). The arrows indicate the direction of the view (through the wall) with respect to the upper panel.

Figure 6 depicts the structural behavior of the concentrated system (*c* = 25.6%). In contrast to the previous system, the associates are relatively compressed, which promotes their spatial arrangement. The top-to-bottom view inside the slits shows the dense arrangement of the micellar centers in planar layers. The perspective views confirm that the *y* and *z* positions of the associates in layers remained random even though the associates were very close to each other.

A comparison of snapshots with the same width and an increasing concentration is of interest. While the numbers of layers in slits with *D* = 10 (one layer) and *D* = 20 and 25 (several layers) did not change with the concentration, for *D* = 15, the number of layers changed from one layer at the low concentration to a sort of “zig-zag” arrangement of the associates in two layers at the high concentration. This, we believe, was caused by a “certain incompatibility” of the optimum size of associates and the (quite narrow) slit width under a fairly strong steric constraint. This slight “anomaly” is discussed in more detail in the following sections.

**Figure 7:**
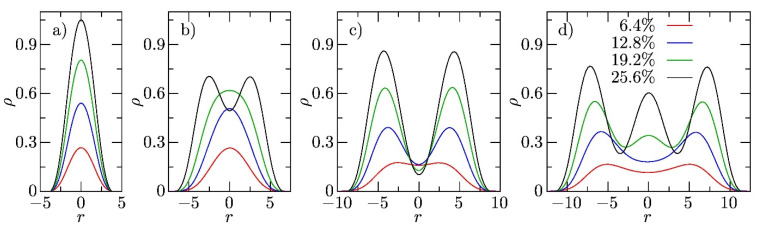
The density profiles of B beads in slits with slightly repulsive walls, *a*_BW_ = 30, and with widths (**a**) *D* = 10, (**b**) D = 15, (**c**) D = 20, and (**d**) D = 25 for several concentrations.

The preferential location of the micelles inside slits with respect to the *x*-axis is proven by the ensemble-average *x*-dependent density of polymer beads (particularly by the density of the core-forming beads B) across the slit. Figure 7a–d depict the density profiles of the insoluble beads in slits differing in width (*D* = 10, 15, 20, and 25). Each frame shows the evolution of the density profiles with increasing concentrations, i.e., four curves for *c* = 6.4, 12.8, 19.2, and 25. 4%.

The frame for *D* = 10 shows monomodal profiles with the maximum in the middle of the slit, confirming the conclusion drawn from the corresponding snapshots. As already mentioned, the concentration-dependent spatial arrangement of the micelles in the slit with *D* = 15 is of interest. The monomodal curves for low concentrations broadened with an increasing concentration, their curvatures close to the maximum were non-negligibly flattened, and at the highest concentration, the density profile changed from a monomodal to a bimodal curve with a fairly narrow distance between the maxima (smaller than the average radius of micelles—see below), which indicates a “zig-zag” arrangement of the micelles in two close coplanar layers in the central part of the slit. The density profiles for *D* = 20 are for all concentrations with fairly symmetric bimodal curves, and those for *D* = 25 are almost symmetric curves (except the slight asymmetry at the lowest concentration caused by low numbers of micelles in the simulation box). The number of layers for *D* = 25 also changed with the concentration (which was not obvious in the snapshots), but the distances between the layers roughly matched the size of associates. With the increase in concentration, significant crowding of the micelles led to more structurally ordered systems that better exploited the available space. This trend can be seen in the narrowing peaks and increased differences between the minima and maxima in the density profiles.

The results of self-assembling systems in slits with inert walls show a weak adsorption of B beads at the wall, but in all other respects are very similar to the results of the slightly repulsive wall, and only selected results of the slit width *D* = 15 are shown in the Supplementary Materials (SMs).

### 3.4. Slightly Attractive Walls

**Figure 8:**
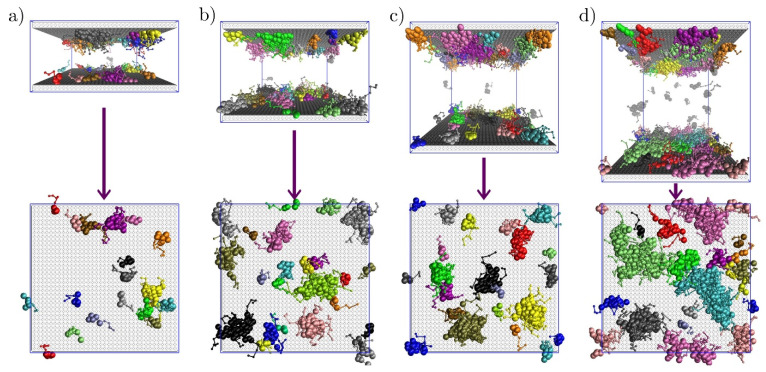
Randomly chosen snapshots of a concentration of 6.4% and slits with slightly attractive walls, *a*_BW_ = 20, and with widths (**a**) *D* = 10, (**b**) *D* = 15, (**c**) *D* = 20, and (**d**) *D* = 25. For clarity, the individual aggregates are labeled by different colors. The B beads are shown as balls and the A beads as thin rods. The aggregates in the slab are grey. The top row provides the views inside the slits in the *z* direction (i.e., rectangular to the *xy* plane), and the bottom row contains the corresponding perspective views through the wall (along the *x*-axis, i.e., rectangular to the *yz* plane). The arrows indicate the direction of the view (through the wall) with respect to the upper panel.

The behavior of associating copolymer systems in the slits with attractive walls differed significantly from that in the slits with inert and slightly repulsive walls. The adsorption on the walls competes with the association process and restricts the formation of non-adsorbed micelles. Figure 8 depicts the behavior of the system in slits with slightly attractive walls at a low concentration, *c* = 6.4%. The upper row of snapshots shows the top-to-bottom views in the slits with increasing *D*. The panels show that almost all chains were adsorbed on the walls, and just a few unimers (sometimes dimers or low associates) coexisted in the middle part of the slit. The lower row shows the chains adsorbed via B segments on the bottom wall. The fraction of the wall surface covered by adsorbed chains was low in the narrow slits because the total amount of the polymer was low. In slits with *D* = 10, the adsorbed species were mainly unimers and very small associates. As the *D* increased, the coverage of the walls increased, and the size of adsorbed associates grew because the slit volume, and consequently the number of chains at a given concentration, increased, while the surface of the walls remained constant.

**Figure 9:**
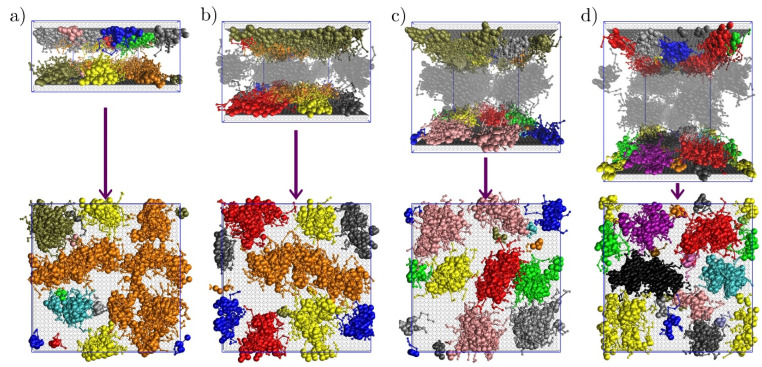
Randomly chosen snapshots of a concentration of 25.6% and slits with slightly attractive walls, *a*_BW_ = 20, with widths of (**a**) *D* = 10, (**b**) *D* = 15, (**c**) *D* = 20, and (**d**) *D* = 25. For clarity, the individual aggregates are labeled by different colors. The B beads are shown as balls, and the A beads as thin rods. The aggregates in the slab are grey. The top row provides the views inside the slits in the *z* direction (i.e., rectangular to the *xy* plane), and the bottom row contains the corresponding perspective views through the wall (along the *x*-axis, i.e., rectangular to the *yz* plane). The arrows indicate the direction of the view (through the wall) with respect to the upper panel.

The snapshots in Figure 9 depict the sorption–association competition at the high concentration, *c* = 25.6%. In the narrow slit with *D* = 10, all chains were adsorbed on the wall in the form of fairly large associates. In broader slits, the coverage of the wall remained approximately the same as that in the narrow slit despite that the number of chains in the slit at a given concentration increased with the *D*. The fact that (i) the excess chains formed the non-adsorbed associates inside the wider slits at elevated concentrations when the coverage of the walls by the adsorbed chains was only partial, (ii) further adsorption of the chains simultaneously stopped, and (iii) the maximum coverage was the same in slits differing in *D* indicate that the competing forces promoting the adsorption and the association were comparable, and none of them were the dominant behavior.

**Figure 10:**
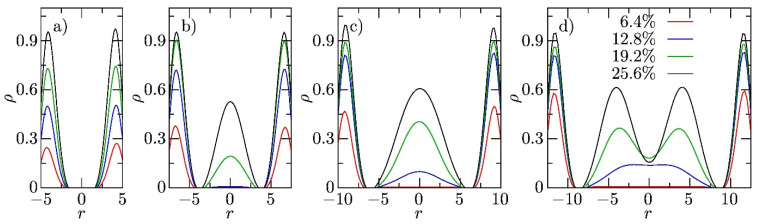
The density profiles of B beads in slits with slightly attractive walls, *a*_BW_ = 20, and with widths of (**a**) *D* = 10, (**b**) *D* = 15, (**c**) *D* = 20, and (**d**) *D* = 25 at several concentrations.

The density profiles in Figure 10 support the conclusion drawn from the above snapshots. A comparison of the areas of peaks close to the walls indicates that the adsorption stopped before the walls were completely covered: the areas of peaks grew at low concentrations with the *D* (see, e.g., the red curves in different frames), but they remained the same (maximum ca. 9.3) at the high concentration, *c* = 25.6%. A comparison of the curves with increasing concentrations in the individual frames in Figure 10 and the plot of the total numbers of adsorbed B beads on the unit wall surface, *ρ*_c_, shown in the Supplementary Materials (SMs), illustrates the saturation effect even better. They confirm the conclusions on the well-balanced interplay of the forces promoting absorption and the forces promoting association. As both forces were equal in strength, their competition resulted in the establishment of mutually inter-related concentration-dependent equilibria, which controlled the balance of both processes.

### 3.5. Strongly Attractive Walls

**Figure 11:**
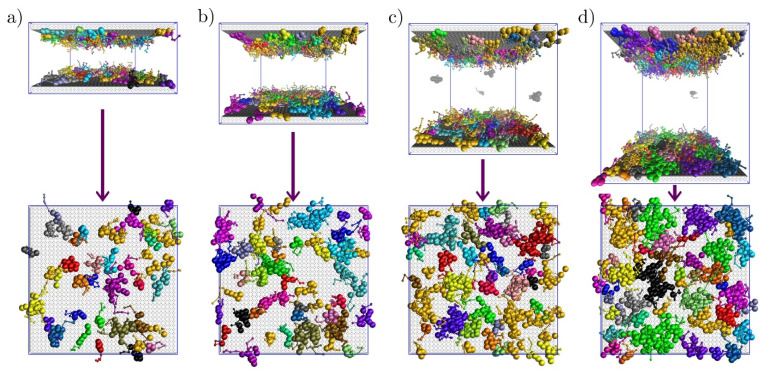
Randomly chosen snapshots of a concentration of 6.4% and slits with strongly attractive walls, *a*_BW_ = 15, and with widths of (**a**) *D* = 10, (**b**) *D* = 15, (**c**) *D* = 20, and (**d**) *D* = 25. For clarity, the individual aggregates are labeled by different colors. The B beads are shown as balls, and the A beads as thin rods. The aggregates in the slab are grey. The top row provides the views inside the slits in the *z* direction (i.e., rectangular to the *xy* plane), and the bottom row contains the corresponding perspective views through the wall (along the *x*-axis, i.e., rectangular to the *yz* plane). The arrows indicate the direction of the view (through the wall) with respect to the upper panel.

**Figure 12:**
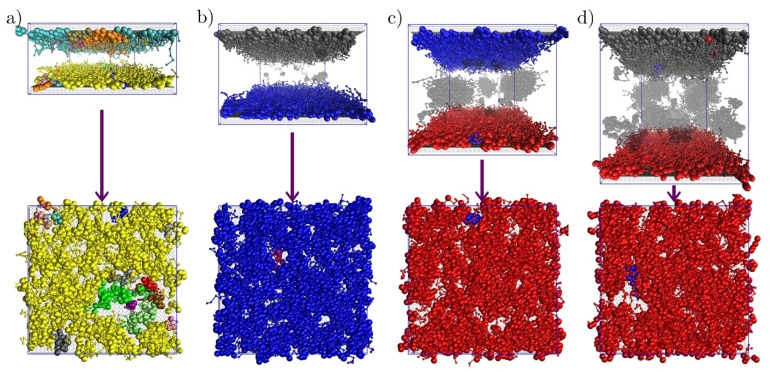
Randomly chosen snapshots of a concentration of 25.6% and slits with strongly attractive walls, *a*_BW_ = 15, and with widths of (**a**) *D* = 10, (**b**) *D* = 15, (**c**) *D* = 20, and (**d**) *D* = 25. For clarity, the individual aggregates are labeled by different colors. The B beads are shown as balls, and the A beads as thin rods. The aggregates in the slab are grey. The top row provides the views inside the slits in the *z* direction (i.e., rectangular to the *xy* plane), and the bottom row contains the corresponding perspective views through the wall (along the *x*-axis, i.e., rectangular to the *yz* plane). The arrows indicate the direction of the view (through the wall) with respect to the upper panel.

The last part of the study was devoted to the slits with strongly attractive walls, which were modeled by the interaction parameter *a*_BW_ = 15. The snapshots in Figure 11 (low concentration, *c* = 6.4%) and Figure 12 (high concentration, *c* = 25.6%) suggest that the adsorption dominated the behavior and significantly restricted the association of non-adsorbed chains in the central parts of the slits. The coverage of the wall was significant even at the low concentration; almost all chains were adsorbed in the form of separate irregular associates and just a few free unimers coexisted in the middle of the slit. The coverage increased with the *D* and the average size of the adsorbed associates grew. The walls of the slit with *D* = 25 were fairly densely covered by fairly large associates.

**Figure 13:**
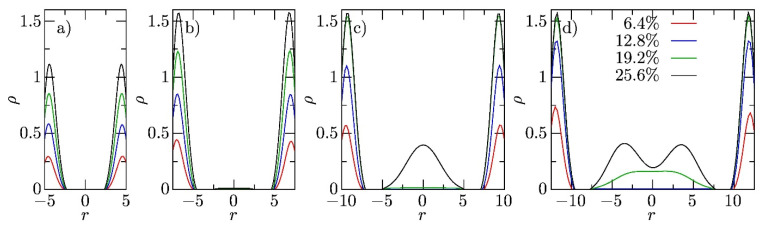
The density profiles of B beads in slits with strongly attractive walls, *a*_BW_ = 15, and with widths of (**a**) *D* = 10, (**b**) *D* = 15, (**c**) *D* = 20, and (**d**) *D* = 25 at several concentrations.

At the high concentration, the wall coverage was almost complete in all slits—even in the narrow one, where we could still identify a few separate islands. In wide slits, both the snapshots and central peaks in the density profiles in Figure 13 indicate that the excess chains formed the non-adsorbed associates. Nevertheless, Figure 13 unambiguously shows that the dissolved associates did not form until the walls were fully and continuously covered (which corresponds to a density of 1.6 of insoluble B beads close to the wall in wide slits). The fairly thick layer of adsorbed chains appreciably reduced the free volume inside the slits where the non-adsorbed associates could form. Therefore, they were strongly crowded and structurally ordered, even though their number was relatively low.

## 4. Conclusions

This paper addresses the behavior of the semi-dilute solutions of self-assembling copolymers in selective solvents submitted to mild steric constraints in slits with either non-attractive or attractive walls. Despite the numerous studies on constrained polymer self-assembly, to the best of our knowledge, this region of medium concentrations and mild constraints has mostly eluded the interest of polymer scientists. The simulation study reveals that associates (spherical core–shell micelles with the admixture of rod-like ellipsoidal core–shell particles) formed in bulk selective solvents at elevated concentrations are partly structurally organized. The organization is imperfect and improves with increasing concentration. The mild steric constraints imposed by medium-wide slits hardly affect the number and size of the associates but promote their structural arrangement in layers parallel to the walls.

While non-attracting walls influence the self-assembly only a little, the effect of attractive walls is significant. The adsorption of polymer chains on slit walls lowers the number of non-adsorbed chains and restricts the self-assembly. In slits with weakly attractive walls, the self-assembly inside the slit efficiently competes with the adsorption. The non-adsorbed micelles start to form inside the slit when the walls are only partly covered by the polymer adsorbed in the form of pancake-like associates and the progressive self-assembly inside the slit prevents further adsorption of copolymers on the walls. This means that mutually inter-related equilibria comprising both the adsorption and formation of non-adsorbed associates control the balance between the copolymer adsorbed on the walls and self-assembled in the solution.

In slits with strongly attractive walls, adsorption dominated the behavior. At lower concentrations, all chains were adsorbed in the form of individual pancake-deformed individual associates, the size of which increased with the number of chains in the slit (i.e., with the concentration and at a given concentration with the slit width). The coverage of the walls increased with an increasing concentration until the walls were densely and continuously covered by copolymer chains adsorbed via B beads. Non-adsorbed micelles started to form only after full wall coverage was reached. The number of non-adsorbed associates was low compared to slits with non-adsorbing or slightly adsorbing walls, but as the walls were covered by a fairly thick layer of the adsorbed copolymer, the free volume in the central part of the slit was thin, which prevented the formation of associates in narrow slits. Nevertheless, non-adsorbed associates formed in wide slits. Their number was fairly low, but they were sterically confined and notably packed in a small volume, which led to their structural organization in layers parallel to the walls.

In the near future, we plan to carry out DPD studies on the behavior of self-assembling copolymer systems in narrow pores, both static and under flow, which should provide essential information for microfluidics.

## Figures and Tables

**Table 1:** Interaction parameters and friction coefficients used in performed simulations.

Interaction Parameter *a_ij_*	Bead A	Bead B	Wall Bead W	Solvent Bead S
Bead A	25	37.5	25	25
Bead B		25	15 20 25 30	37.5
Wall bead W			25	25
Solvent bead S				25

## Data Availability

The data presented in this study are available upon request from the corresponding authors.

## Supplementary Materials

The following supporting information can be downloaded at: https://www.mdpi.com/article/10.3390/polym15224458/s1. Reference [64] is cited in the Supplementary Material S1. Imperfect structural arrangement of aggregates in semi-dilute bulk solutions; S2. The proof that the studied systems are not kinetically frozen; S3. Slightly unusual behavior of systems in slits with D = 15; S4. Selected results for slits with inert walls; S5. Total numbers of B beads adsorbed on slightly and strongly attractive walls; Figure S1. Left: the density profiles of solvophobic block B (full lines) in the x direction (first pic-ture), y direction (second), and z direction (third). Right: density profiles of solvophilic block A (dashed lines) in the x direction (first picture), y direction (second), and z direction (third). Blue curve corresponds to c = 6.4%, magenta to c = 12.8%, orange to c = 19.2%, green to c = 22 %, yel-low to c = 25.6%, purple to c = 27%, and cyan to c = 32; Figure S2. (a) Fluctuations of the number-weighted average aggregation numbers for different polymer concentrations (oligomers excluded); (b) oscillations of the number of aggregates during the simulation run for dilute, semi-dilute, and concentrated bulk solutions (oligomers excluded (As ≥ 10)); Figure S3. The weight distribution functions of association numbers of micelles formed in slits with slightly repulsive walls, aBW = 30, and differing in D at (a) c = 12.8 vol.% and (b) c = 25.6 vol.%; Figure S4. Randomly chosen snapshots of aggregates for concentrations (a) c = 12.8 vol.% and (b) c = 25.6 vol.%. and slits with inert walls, aBW = 25, and with widths D = 15. %. For clarity, the in-dividual aggregates are labeled by different colors. The B beads are shown as balls and A beads as thin rods. The top row provides the views inside the slits in the z direction (i.e., rectangular to the xy plane), and the bottom row contains the corresponding perspective views through the wall (along the x axis, i.e., rectangular to the yz plane). The arrows indicate the direction of the view (through the wall) with respect to the upper panel. (c) The corresponding density profiles; Figure S5. The dependences of the number of B beads adsorbed on unit surface, *ρ*_c_, on concentra-tion, c, in slit with slightly attractive walls, aBW = 20, for several slit widths; Figure S6. The dependences of the number of B beads adsorbed on unit surface, *ρ*_c_, on concentra-tion, c, in slit with strongly attractive wall, aBW = 15, for several slit widths.

## Abbreviations

The following abbreviations are used in this manuscript:

DPD	Dissipative particle dynamics
EICA	Evaporation-induced confinement assembly
FENE	Finitely extendible non-linear elastic
FH	Flory–Huggins

## References

[1] Nagarajan R. (2017). Constructing a molecular theory of self-assembly: Interplay of ideas from surfactants and block copolymers. Adv. Colloid Interface Sci..

[2] Nagarajan R., Ganesh K. (1989). Block copolymer self-assembly in selective solvents–spherical micelles with segregated cores. J. Chem. Phys..

[3] Syamala P.P.N., Wurthner F. (2020). Modulation of the Self-Assembly of pi-Amphiphiles in Water from Enthalpy- to Entropy-Driven by Enwrapping Substituents. Chem. A Eur. J..

[4] Zheng B., Yu L.L., Dong H.Z., Zhu J.M., Yang L., Yuan X.S. (2022). Photo-Responsive Micelles with Controllable and Co-Release of Carbon Monoxide, Formaldehyde and Doxorubicin. Polymers.

[5] Avsar S.Y., Kyropoulou M., Di Leone S., Schoenenberger C.A., Meier W.P., Palivan C.G. (2019). Biomolecules Turn Self-Assembling Amphiphilic Block Co-polymer Platforms Into Biomimetic Interfaces. Front. Chem..

[6] Kataoka K., Harada A., Nagasaki Y. (2012). Block copolymer micelles for drug delivery: Design, characterization and biological significance. Adv. Drug Deliv. Rev..

[7] Blanazs A., Armes S.P., Ryan A.J. (2009). Self-Assembled Block Copolymer Aggregates: From Micelles to Vesicles and their Biological Applications. Macromol. Rapid Commun..

[8] Kabanov A.V., Alakhov V.Y. (2002). Pluronic (R) block copolymers in drug delivery: From micellar nanocontainers to biological response modifiers. Crit. Rev. Ther. Drug Carr. Syst..

[9] Chrysostomou V., Forys A., Trzebicka B., Demetzos C., Pispas S. (2022). Amphiphilic Copolymer-Lipid Chimeric Nanosystems as DNA Vectors. Polymers.

[10] Huang C.W., Chang Y.Y., Cheng C.C., Hung M.T., Mohamed M.G. (2022). Self-Assembled Supramolecular Micelles Based on Multiple Hydrogen Bonding Motifs for the Encapsulation and Release of Fullerene. Polymers.

[11] Perumal S., Atchudan R., Lee W. (2022). A Review of Polymeric Micelles and Their Applications. Polymers.

[12] Hils C., Manners I., Schöbel J., Schmalz H. (2021). Patchy Micelles with a Crystalline Core: Self-Assembly Concepts, Properties, and Applications. Polymers.

[13] Xiao L.L., Zhou X., Yue K., Guo Z.H. (2021). Synthesis and Self-Assembly of Conjugated Block Copolymers. Polymers.

[14] Gohy J.-F., Zhao Y. (2013). Photo-responsive block copolymer micelles: Design and behavior. Chem. Soc. Rev..

[15] Walther A., Mueller A.H.E. (2013). Janus Particles: Synthesis, Self-Assembly, Physical Properties, and Applications. Chem. Rev..

[16] Mai Y.Y., Eisenberg A. (2012). Self-assembly of block copolymers. Chem. Soc. Rev..

[17] Moughton A.O., Hillmyer M.A., Lodge T.P. (2012). Multicompartment Block Polymer Micelles. Macromolecules.

[18] O’Reilly R.K., Hawker C.J., Wooley K.L. (2006). Cross-linked block copolymer micelles: Functional nanostructures of great potential and versatility. Chem. Soc. Rev..

[19] Uchman M., Stepanek M., Prevost S., Angelov B., Bednar J., Appavou M.S., Gradzielski M., Prochazka K. (2012). Coassembly of Poly(ethylene oxide)-block-poly(methacrylic acid) and N-Dodecylpyridinium Chloride in Aqueous Solutions Leading to Ordered Micellar Assemblies within Copolymer Aggregates. Macromolecules.

[20] Podhajecka K., Stepanek M., Prochazka K., Brown W. (2001). Hybrid polymeric micelles with hydrophobic cores and mixed polyelectrolyte/nonelectrolyte shells in aqueous media. 2. Studies of the shell behavior. Langmuir.

[21] Matejicek P., Uhlik F., Limpouchova Z., Prochazka K., Tuzar Z., Webber S. (2002). Experimental study of hydrophobically modified amphiphilic block copolymer micelles using light scattering and nonradiative excitation energy transfer. Macromolecules.

[22] Plestil J., Kriz J., Tuzar Z., Prochazka K., Melnichenko Y.B., Wignall G.D., Talingting M.R., Munk P., Webber S.E. (2001). Small-angle neutron scattering study of onion-type micelles. Macromol. Chem. Phys..

[23] Wong C.K., Qiang X., Müller A.H., Gröschel A.H. (2020). Self-Assembly of block copolymers into internally ordered microparticles. Prog. Polym. Sci..

[24] Ok S., Vayer M., Sinturel C. (2021). A decade of innovation and progress in understanding the morphology and structure of heterogeneous polymers in rigid confinement. Soft Matter.

[25] Tong L., Nabae Y., Hirai T., Yabu H., Hayakawa T. (2023). Creation of Thermal Response Ordered Mesostructure Polymer Particles Using Diblock Copolymers via 3D Confined Self-Assembly. Macromol. Chem. Phys..

[26] Juan Y.T., Lai Y.F., Li X.Y., Tai T.C., Huang C.F., Lin C.H., Li B.H., Shi A.C., Hsueh H.Y. (2023). Self-Assembly of Gyroid-Forming Diblock Copolymers under Spherical Confinement. Macromolecules.

[27] Ashwini T., Narayan R., Shenoy P.A., Nayak U.Y. (2022). Computational modeling for the design and development of nano based drug delivery systems. J. Mol. Liq..

[28] Peng M.L., Hu D.W., Chang X.H., Zhu Y.T. (2022). Confined Self-Assembly of Block Copolymers within Emulsion Droplets: A Perspective. J. Phys. Chem. B.

[29] Lu Z., Liu G., Liu F. (2001). Block copolymer microspheres containing intricate nanometer-sized segregation patterns. Macromolecules.

[30] Wu Y., Wang K., Tan H., Xu J., Zhu J. (2017). Emulsion solvent evaporation-induced self-assembly of block copolymers containing pH-sensitive block. Langmuir.

[31] Ku K.H., Shin J.M., Klinger D., Jang S.G., Hayward R.C., Hawker C.J., Kim B.J. (2016). Particles with tunable porosity and morphology by controlling interfacial instability in block copolymer emulsions. ACS Nano.

[32] Wang X., Limpouchova Z., Prochazka K., Raya R.K., Min Y.G. (2022). Modeling the Phase Equilibria of Associating Polymers in Porous Media with Respect to Chromatographic Applications. Polymers.

[33] Liu Y.L., Ke F.F., Li Y.C., Shi Y., Zhang Z., Chen Y.M. (2023). Emulsion confined block copolymer self-assembly: Recent progress and prospect. Nano Res..

[34] Mendoza C., Nirwan V.P., Fahmi A. (2023). Nanofabrication of hybrid nanomaterials: Macroscopically aligned nanoparticles pattern via directed self-assembly of block copolymers. J. Appl. Polym. Sci..

[35] Cifra P., Bleha T. (2021). Piston Compression of Semiflexible Ring Polymers in Channels. Macromol. Theory Simul..

[36] Mijangos C., Hernandez R., Martin J. (2016). A review on the progress of polymer nanostructures with modulated morphologies and properties, using nanoporous AAO templates. Prog. Polym. Sci..

[37] Binder K., Milchev A. (2012). Polymer brushes on flat and curved surfaces: How computer simulations can help to test theories and to interpret experiments. J. Polym. Sci. Part B-Polym. Phys..

[38] Binder K., Kreer T., Milchev A. (2011). Polymer brushes under flow and in other out-of-equilibrium conditions. Soft Matter.

[39] Viduna D., Limpouchova Z., Prochazka K. (2001). Monte Carlo simulation of polymer brushes in narrow pores. J. Chem. Phys..

[40] Limpouchova Z., Viduna D., Prochazka K. (1997). Mixed systems of tethered chains in spherical volumes. A model for cores of mixed copolymer micelles. Macromolecules.

[41] Wang X., Procházka K., Limpouchová Z. (2020). Partitioning of polymers between bulk and porous media: Monte Carlo study of the effect of pore size distribution. J. Colloid Interface Sci..

[42] Wang X., Prochazka K., Limpouchova Z. (2019). Pore size effect on the separation of polymers by interaction chromatography. A Monte Carlo study. Anal. Chim. Acta.

[43] Wang X., Limpouchova Z., Prochazka K. (2019). Separation of polymers differing in their chain architecture by interaction chromatography: Phase equilibria and conformational behavior of polymers in strongly adsorbing porous media. Polymer.

[44] Ahn J., Chang T., Wang X., Limpouchova Z., Prochazka K. (2017). Influence of the chain architecture and the presence of end-groups or branching units chemically different from repeating structural units on the critical adsorption point in liquid chromatography. Macromolecules.

[45] Cifra P., Bleha T. (2005). Confined macromolecules in polymer materials and processes. New Polym. Mater..

[46] Skrinarova Z., Bleha T., Cifra P. (2002). Concentration effects in partitioning of macromolecules into pores with attractive walls. Macromolecules.

[47] Hou C.L., Gao L.J., Wang Y.M., Yan L.T. (2022). Entropic control of nanoparticle self-assembly through confinement. Nanoscale Horiz..

[48] Huang J.-H., Wu J.-J., Huang X.-W. (2016). Self-assembly of symmetric rod-coil diblock copolymers in cylindrical nanopore. RSC Adv..

[49] Deng R.H., Li H., Liang F.X., Zhu J.T., Li B.H., Xie X.L., Yang Z.Z. (2015). Soft Colloidal Molecules with Tunable Geometry by 3D Confined Assembly of Block Copolymers. Macromolecules.

[50] Hoogerbrugge P.J., Koelman J.M.V.A. (1992). Simulating microscopic hydrodynamic phenomena with dissipative particle dynamics. Europhys. Lett..

[51] Espanol P., Warren P.B. (2017). Perspective: Dissipative particle dynamics. J. Chem. Phys..

[52] Gonzalez-Melchor M., Mayoral E., Velazquez M.E., Alejandre J. (2006). Electrostatic interactions in dissipative particle dynamics using the Ewald sums. J. Chem. Phys..

[53] Groot R.D., Karttunen M., Vattulainen I., Lukkarinen A. (2004). Applications of dissipative particle dynamics. Novel Methods in Soft Matter Simulations.

[54] Groot R.D. (2003). Electrostatic interactions in dissipative particle dynamics–simulation of polyelectrolytes and anionic surfactants (vol 118, pg 11265, 2003). J. Chem. Phys..

[55] Groot R.D., Warren P.B. (1997). Dissipative particle dynamics: Bridging the gap between atomistic and mesoscopic simulation. J. Chem. Phys..

[56] Espanol P., Warren P. (1995). Statistical-mechanics of dissipative particle dynamics. Europhys. Lett..

[57] Zhao J.Y., Chen S., Zhang K.X., Liu Y. (2021). A review of many-body dissipative particle dynamics (MDPD): Theoretical models and its applications. Phys. Fluids.

[58] Lombardo D., Kiselev M.A., Magazù S., Calandra P. (2015). Amphiphiles Self-Assembly: Basic Concepts and Future Perspectives of Supramolecular Approaches. Adv. Condens. Matter Phys..

[59] Blovský T., Šindelka K., Limpouchová Z., Procházka K. (2022). Changes in Ion Concentrations upon the Binding of Short Polyelectrolytes on Phospholipid Bilayers: Computer Study Addressing Interesting Physiological Consequences. Polymers.

[60] Prochazka K., Limpouchova Z., Stepanek M., Sindelka K., Lisal M. (2022). DPD Modelling of the Self- and Co-Assembly of Polymers and Polyelectrolytes in Aqueous Media: Impact on Polymer Science. Polymers.

[61] Sindelka K., Limpouchova Z., Prochazka K. (2021). Solubilization of Charged Porphyrins in Interpolyelectrolyte Complexes: A Computer Study. Polymers.

[62] Sindelka K., Limpouchova Z., Lisal M., Prochazka K. (2016). The electrostatic co-assembly in non-stoichiometric aqueous mixtures of copolymers composed of one neutral water-soluble and one polyelectrolyte (either positively or negatively charged) block: A dissipative particle dynamics study. Phys. Chem. Chem. Phys..

[63] Groot R.D., Rabone K.L. (2001). Mesoscopic simulation of cell membrane damage, morphology change and rupture by nonionic surfactants. Biophys. J..

[64] Rubinstein M., Colby R.H. (2003). Polymer Physics.

[65] Sindelka K., Lisal M. (2021). Interplay between surfactant self-assembly and adsorption at hydrophobic surfaces: Insights from dissipative particle dynamics. Mol. Phys..

[66] Petrus P., Lisal M., Brennan J.K. (2010). Self-Assembly of Lamellar- and Cylinder-Forming Diblock Copolymers in Planar Slits: Insight from Dissipative Particle Dynamics Simulations. Langmuir.

[67] Posel Z., Posocco P., Lisal M., Fermeglia M., Pricl S. (2016). Highly grafted polystyrene/polyvinylpyridine polymer gold nanoparticles in a good solvent: Effects of chain length and composition. Soft Matter.

[68] Seaton M.A. (2021). DL_MESO_DPD: Development and use of mesoscale modelling software. Mol. Simul..

[69] Prinsen P., Warren P.B., Michels M.A.J. (2002). Mesoscale simulations of surfactant dissolution and mesophase formation. Phys. Rev. Lett..

[70] Milchev A., Bhattacharya A., Binder K. (2001). Formation of block copolymer micelles in solution: A Monte Carlo study chain length dependence. Macromolecules.

[71] Balastre M., Li F., Schorr P., Yang J.C., Mays J.W., Tirrell M.V. (2002). A study of polyelectrolyte brushes formed from adsorption of amphiphilic diblock copolymers using the surface forces apparatus. Macromolecules.

