# Supplementary material for: Self-Assembly of Symmetric Copolymers in Slits with Inert and Attractive Walls
Source: Polymers (Basel). 2023 Nov 18;15(22):4458. doi: 10.3390/polym15224458 (PMC10675682; doi:10.3390/polym15224458)
Supplement: Supplementary file 1 [file polymers-15-04458-s001.zip › polymers-2657316-supplementary.pdf]

Supplementary Materials

S1. Imperfect structural arrangement of aggregates in semi-dilute bulk solutions

We investigated if and how the elevated concentration affects the organization of aggregates in bulk solutions. It is a well-known fact that the lamellar structure is a typical organization of the melt of symmetric high-molar-mass diblock copolymers [1]. The aggregates in studied solutions are fairly crowded, but their concentrations are far from that in the melt. Therefore, it is interesting to study the onset and gradual development of the concentration-dependent organization of associates in space. As the formation of organized structures (lamellas, hexagonal phase, or other structural motifs, both perfect and imperfect) generates the oscillations in local density of polymer blocks, the scans of the average density of individual blocks in properly selected directions should reveal the spatial orientation. The problem is that the structural motifs in bulk can be oriented in any direction. Hence, we evaluated the density profiles of insoluble (full lines) and soluble (dashed lines) blocks at different concentrations along all three axes, x, y, and z, and in Fig. S1 we outline the results elucidating the behavior.

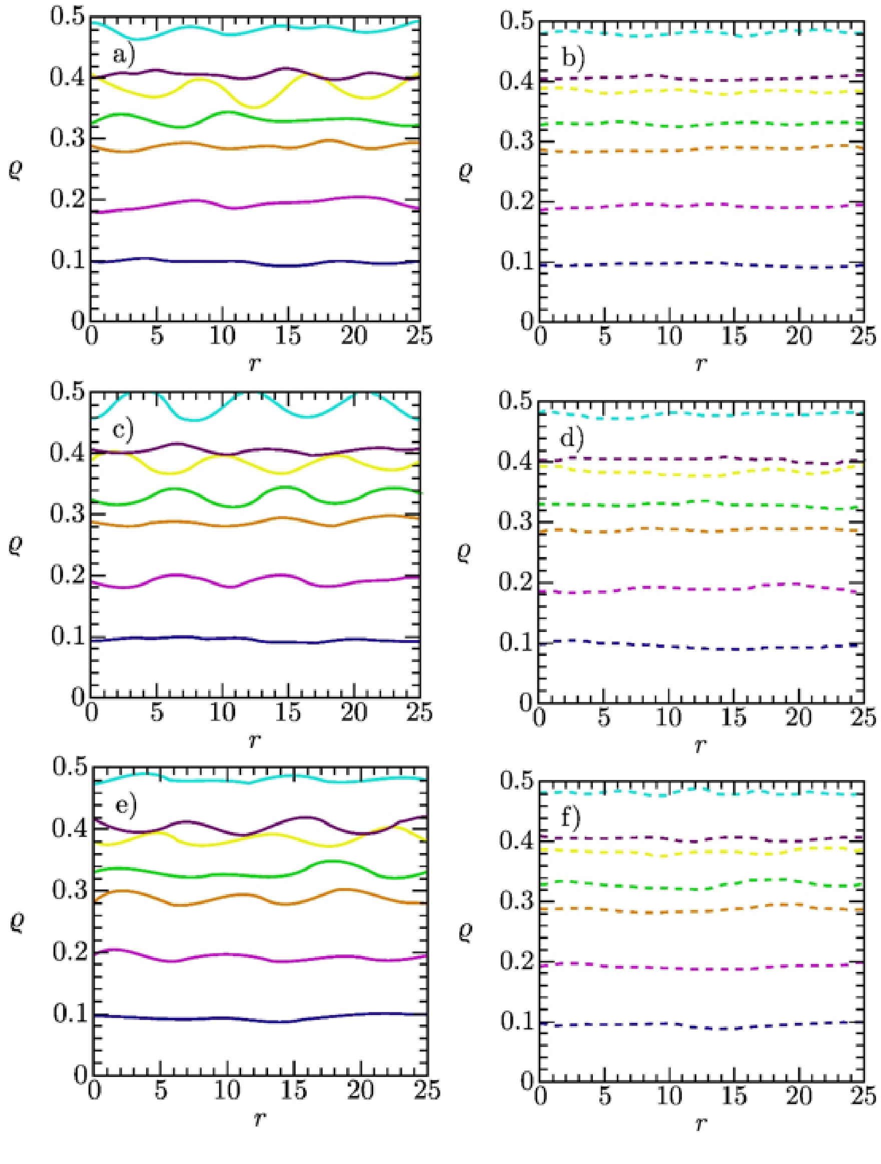

Figure S1. Left: the density profiles of solvophobic block B (full lines) in the x direction (first picture), y direction (second), and z direction (third). Right: density profiles of solvophilic block A (dashed lines) in the x direction (first picture), y direction (second), and z direction (third). Blue

curve corresponds to c = 6.4%, magenta to c = 12.8%, orange to c = 19.2%, green to c = 22 %, yellow to c = 25.6%, purple to c = 27%, and cyan to c = 32.

The fact that both densities are constant at low concentrations and the density of insoluble blocks (averaged over the planes perpendicular to the selected directions) oscillates at higher concentrations while that of soluble blocks remains constant indicates the preferential localization of micellar cores in planar layers. The picture is only qualitative because the amplitudes of oscillations depend on the random orientation of layers in the simulation box and the average density does not provide information on the potential organization of cores in layers. Nevertheless, the quintessence of the message extracted from Fig. S1 is clear and confirms the conclusion on the imperfect spatial organization of associates at elevated concentrations.

S2. The proof that the studied systems are not kinetically frozen

From the simulation viewpoint, the structural organization implies the danger that the crowded systems could freeze in arrested states. To prove that the system does not freeze, below we plot the fluctuations of association numbers of associates (excluding the unimers) for the low, medium, and high concentrations. The plots show fast fluctuations, and the changes represent 10 (in some cases almost 20) units. Because the simulation box contains several associates (tens of associates at high concentrations), we plotted the instantaneous number-average $\langle A_S \rangle_n$ values for associates formed in individual time steps in the simulation box. This means that the presented quantity fluctuates less than the A_S of individual associates. The highest and lowest A_S values are missing as a result of the averaging of tents of fluctuating values. Nevertheless, the plots in Fig. S2 unambiguously show that none of the systems freeze during the simulation.

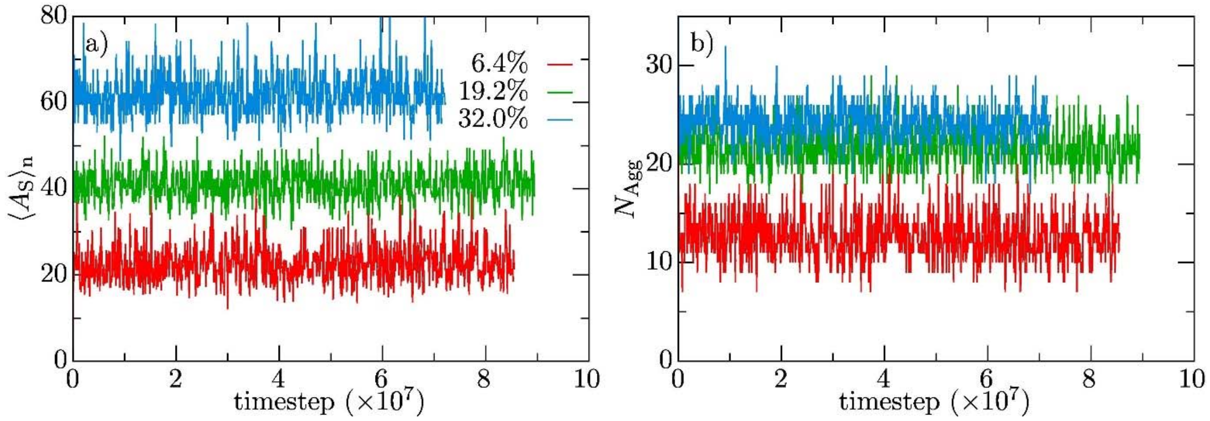

Figure S2. (a) Fluctuations of the number-weighted average aggregation numbers for different polymer concentrations (oligomers excluded); (b) oscillations of the number of aggregates during the simulation run for dilute, semi-dilute, and concentrated bulk solutions (oligomers excluded ($A_s \geq 10$)).

S3. Slightly unusual behavior of systems in slits with D = 15

An important observation is that the system in the slit with D = 15 behaves slightly differently from other systems, presumably as a result of a certain "incompatibility" of the optimum size of micelles and the fairly narrow slit width. This hypothesis assumes that the solvent quality plays the primary role and controls the self-assembly, and the mild steric constraints in slits with non-adsorbing walls play the secondary role. To support this working hypothesis, we present the weight distribution functions of association numbers of micelles formed in slits differing in D at c = 12.8 vol.% in Fig. S3a, and in Fig. S3b, we present analogous curves for c = 25.6 vol.%. The comparison of individual curves and their comparison with the weight distribution of micelles in bulk (Fig. 1 in the mail document) show that the curves are not identical but do not differ considerably from each other, and the effects of concentration and the slit width are relatively mild,

except for the concentration effect on the system with $D = 15$. In this case, the distribution of A_S shifts significantly to higher values with increasing concentration, and the curve develops an important tail towards a high A_S (much more pronounced than in other slits), which reflects the presence of high numbers of large prolate "zig-zag"-arranged associates in two close layers (see Figs. 6 and 7 in the main document).

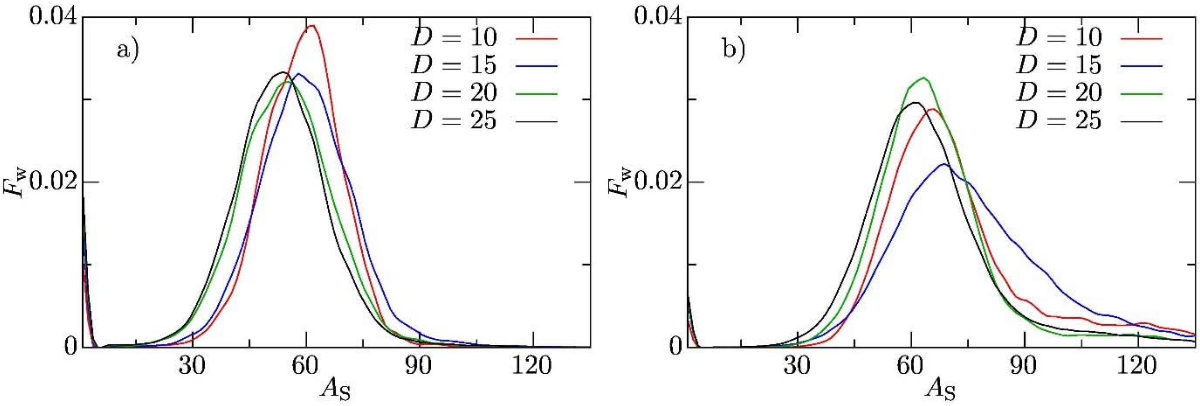

Figure S3. The weight distribution functions of association numbers of micelles formed in slits with slightly repulsive walls, $a_{BW} = 30$, and differing in D at (a) c = 12.8 vol.% and (b) c = 25.6 vol.%.

We believe that the above-outlined simulation results and their analysis explain the slightly unexpected (irregular) behavior of the systems in the slit with $D = 15$. The irregularity obviously assumes that the slit is only slightly wider than the size of associates, and we would like to remind the reader that the observed behavior depends on the parameters used in our model, and in systems differing in the length of A and B blocks or described by different interaction parameters, the irregular behavior can arise in different slit widths because of the different sizes of associates.

S4. Selected results for slits with inert walls

The results for slits with inert walls show a weak adsorption of B beads at the wall, but they are very similar to the results for the slightly repulsive wall, and therefore we present only selected data for the slit width $D = 15$. In Fig. S4, we depict density profiles for the slit width D=15 and corresponding snapshots for two concentrations.

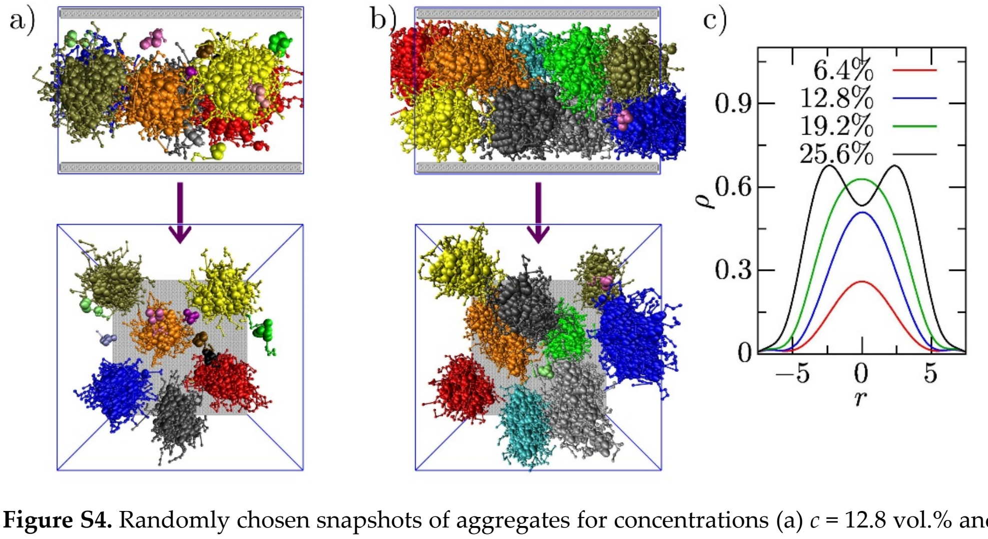

Figure S4. Randomly chosen snapshots of aggregates for concentrations (a) $c = 12.8$ vol.% and (b) $c = 25.6$ vol.%. and slits with inert walls, $a_{BW} = 25$, and with widths $D = 15$. %. For clarity, the individual aggregates are labeled by different colors. The B beads are shown as balls and A beads as thin rods. The top row provides the views inside the slits in the z direction (i.e., rectangular to the xy plane), and the bottom row contains the corresponding perspective views through the wall

(along the x axis, i.e., rectangular to the yz plane). The arrows indicate the direction of the view (through the wall) with respect to the upper panel. (c) The corresponding density profiles.

S5. Total numbers of B beads adsorbed on slightly and strongly attractive walls

To support the conclusions drawn from Fig. 15, we plot the total numbers of adsorbed B beads on the unit wall surface, ρ_c, in Fig. S5. They illustrate the saturation effect even better. They confirm the conclusions on the competitive interplay of forces promoting the absorption and forces promoting the association. As both forces are well-balanced in strength, their competition results in the establishment of mutually interrelated concentration-dependent equilibria that control the balance of both processes.

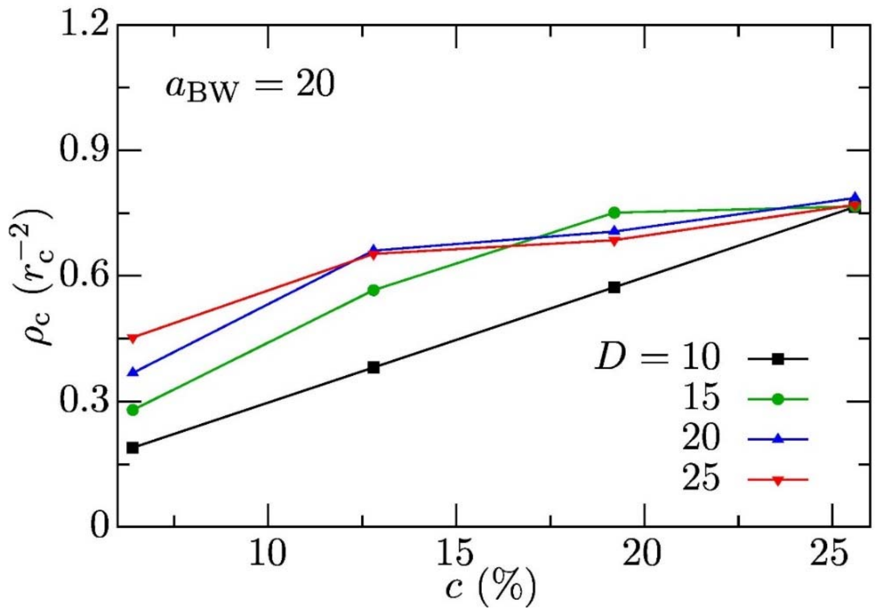

Figure S5. The dependences of the number of B beads adsorbed on unit surface, ρ_c, on concentration, c, in slit with slightly attractive walls, $a_{BW} = 20$, for several slit widths.

Fig. S6 depicts the concentration-dependent number of B beads adsorbed on the unit surface of the strongly attractive wall (curves analogous to those depicted in Fig. S5). In this case, the coverage is according to the expected high. In narrow slits, it linearly increases with polymer concentration. In wide slits (containing important numbers of polymer chains), the curves level off, but this happens after the slit walls are densely covered by adsorbed B beads.

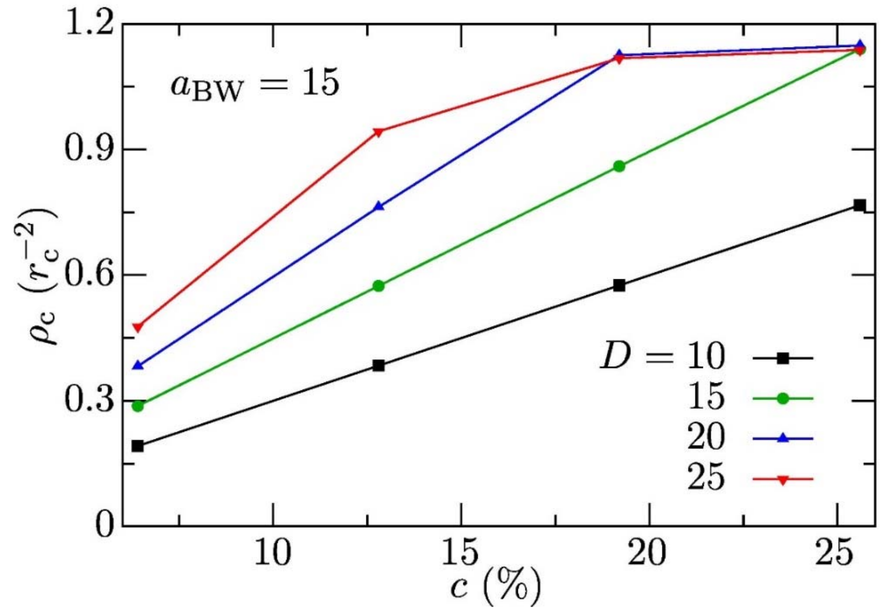

Figure S6. The dependences of the number of B beads adsorbed on unit surface, ρ_c, on concentration, *c,* in slit with strongly attractive wall, $a_{BW} = 15$, for several slit widths.

References:

1. Rubinstein, M.; Colby, R.H. *Polymer Physics*; New York: Oxford University: 2003; p. 440.
